# Heading Estimation for Indoor Pedestrian Navigation Using a Smartphone in the Pocket

**DOI:** 10.3390/s150921518

**Published:** 2015-08-28

**Authors:** Zhi-An Deng, Guofeng Wang, Ying Hu, Di Wu

**Affiliations:** School of Information Science and Technology, Dalian Maritime University, Dalian 116026, China; E-Mails: wangguofeng@dlmu.edu.cn (G.W.); huying@dlmu.edu.cn (Y.H.); wudi@dlmu.edu.cn (D.W.)

**Keywords:** indoor navigation, heading estimation, rotation matrix, principal component analysis, smartphone sensors

## Abstract

Heading estimation is a central problem for indoor pedestrian navigation using the pervasively available smartphone. For smartphones placed in a pocket, one of the most popular device positions, the essential challenges in heading estimation are the changing device coordinate system and the severe indoor magnetic perturbations. To address these challenges, we propose a novel heading estimation approach based on a rotation matrix and principal component analysis (PCA). Firstly, through a related rotation matrix, we project the acceleration signals into a reference coordinate system (RCS), where a more accurate estimation of the horizontal plane of the acceleration signal is obtained. Then, we utilize PCA over the horizontal plane of acceleration signals for local walking direction extraction. Finally, in order to translate the local walking direction into the global one, we develop a calibration process without requiring noisy compass readings. Besides, a turn detection algorithm is proposed to improve the heading estimation accuracy. Experimental results show that our approach outperforms the traditional uDirect and PCA-based approaches in terms of accuracy and feasibility.

## 1. Introduction

Though various indoor pedestrian navigation systems, such as WiFi localization [1], ultra-wideband [2] and radio frequency identification (RFID) [3], have been developed, how to achieve accurate and seamless navigation at low cost is still a challenging task. Most existing technologies depend on some form of dedicated infrastructure, which is expensive for large scale deployment and always not continuously available during pedestrian walking. Recently, a pedestrian dead reckoning (PDR) system [4] using a smartphone has been considered as a promising solution to seamless indoor navigation. The smartphone plays an indispensable role in our daily lives and can be carried almost everywhere we go. This, coupled with the fact that inertial sensors are typically installed in even the low-cost smartphones, has made smartphones ideal devices for continuous indoor navigation.

A central problem for PDR using a smartphone [5] is the user heading estimation. When the user heading and related walking distance per step are obtained, the user location can be determined by computing relative displacement, starting from the initial location that is assumed to be known. If the heading estimation problem is solved well, it can also benefit many other application areas. Particularly, the user heading may be transferred into the user facing direction, which is a critical component in augmented reality, social activities, and human computer interactions [6].

Existing heading estimation approaches [7,8,9] using smartphone inertial sensors always assume that the heading misalignment between device forward direction and the user heading remains constant. Thus, the user heading can be directly determined by device attitude estimation once the offset is known. The assumption is true when the user holds the smartphone in hand or against the ear during phone calling. However, for a smartphone placed in a pocket [10], the yaw angle may change dynamically even when the pedestrian is walking straight, thus rending previous heading estimation approaches inapplicable. Practically, it is more convenient and realistic to enable user acceptance of PDR by assuming a more natural device positions, such as in the pocket. Thus, this work focuses on a smartphone put in a pant pocket, since it is likely the most popular wearing positions, especially for young males [11]. 

Recently, principal component analysis (PCA) has been utilized for heading estimation by exploiting the acceleration signal patterns in the horizontal plane. Though reasonable accuracy performance has been reported in these experiments, some critical problems still need to be addressed to enhance accuracy and feasibility. Firstly, it is a challenging task to obtain the horizontal plane of acceleration signals, since the local device coordinate system may vary all the time while a pedestrian is walking. When placed in a pant pocket, the smartphone swings with the femur bone, continuously altering the device coordinate system. Secondly, even if the local walking direction is accurately extracted in the local device coordinate system, translating it into the global one is still a challenge. The PCA-based approach implements this translation by deploying a compass, which suffers from severe magnetic perturbations in modern man-made structures [12] and is unavailable for most indoor environments.

In order to overcome the aforementioned challenges, we present a novel heading estimation approach based on Rotation Matrix and Principal Component Analysis (RMPCA). The proposed RMPCA may achieve accurate user heading estimation, regardless of the smartphone orientation and placement within the pocket. Our work makes the following three contributions: 

First, to adapt the changing device coordinate system and achieve an accurate estimation of the horizontal plane of acceleration signals, we project all the acceleration signals into a reference coordinate system using a related rotation matrix. The rotation matrix is computed by an extended Kalman filter (EKF)-based attitude estimation model. 

Second, after extracting the local walking direction by utilizing PCA over the horizontal plane, we develop a calibration process to translate the local walking direction into the global one. Compared with the traditional approaches, the major advantage of our calibration process is that we avoid introducing the noisy compass, which is almost always unavailable in most indoor environments. 

Third, we propose a turn detection algorithm to improve the heading estimation accuracy. PCA for local walking direction extraction suffers from significant performance degradation when a user turn occurs. Thus, we deploy relative heading change for heading estimation once a user turn is detected.

In the rest of this paper, we first discuss the related works in Section 2. Section 3 introduces an overview and some key concepts of the proposed approach. Section 4 describes the approach in detail. The experimental results are reported and discussed in Section 5. Conclusions and our future works are presented in the last section. 

## 2. Related Works

Many existing heading estimation approaches for PDR deploy wearable sensors fixed on the human body. In [13], zero velocity updates (ZUPTs) are introduced into the EKF to achieve a reliable user heading estimation when foot-mounted sensors are used. In [14], the user heading is obtained by computing the locations of two infrared ray receiver modules mounted on the subject’s shoulders. In [15], stereo cameras attached on the user’s chest are deployed for heading estimation during pedestrian navigation. Compared with the unconstrained wearable sensors, those carefully attached on fixed human positions always provide more accurate and robust heading estimation results. This is because not only some more extra information such as ZUPT can be exploited, but also the heading misalignment between device forward direction and user heading is always constant during pedestrian walking. However, carrying dedicated devices, which are typically useless for users in their daily lives, with a fixed position for long duration makes them impractical for PDR applications. For the mass market, it is more practical and less intrusiveness to deploy the pervasively available smartphone as a common device for heading estimation.

Most existing smartphone-based user heading estimation approaches [16,17] are based on the highly simplified assumption that the heading misalignment between device forward direction and the user heading remains constant. For a hand-held smartphone, the assumption is true, since the forward device direction is always the same as the user heading. Thus, traditional attitude estimation techniques such as Kalman filter [18] or its variants may be directly applied to achieve reliable heading estimation. However, for a smartphone freely placed in other positions, the assumption is seriously corrupted by the changing device coordinate system. As a result, the smartphone attitude estimation is insufficient for heading estimation, since the misalignment angle may vary with body locomotion.

Recently, studies [5,19] have demonstrated that the positions of the device on the user’s body can be successfully detected by analyzing acceleration patterns generated during walking locomotion. Therefore, it is feasible to determine the user heading by assuming the known device placements. Kunze *et al.* [20] have deployed PCA for walking direction extraction with the device placed in the user’s pant pocket. They first obtain the horizontal plane of acceleration signals by related vertical acceleration component, which is the acceleration signal when the user is approximately static. Then, the local walking direction at device coordinate system is extracted by applying PCA, and finally translated into the global walking direction by a digital compass. Steinhoff *et al.* [21] have conducted an experimental study of such PCA-based techniques, and further improves the heading estimation accuracy by properly filtering acceleration signals. However, the PCA based approach is prone to the inaccurate estimation of horizontal acceleration plane caused by changing device coordinates. Hoseinitabatabaei *et al.* [22] have proposed an uDirect approach to extract the user heading directly within a specific region, where the forward acceleration dominates the horizontal acceleration components. Unfortunately, such a region is always corrupted by sideway acceleration components according to our experimental study. More importantly, neither the PCA nor the uDirect approach can be used indoors, because they both rely on a compass to translate the local direction into the global one, which is almost always unavailable due to the serve indoor magnetic perturbations [23,24].

This work proposes a novel RMPCA approach for user heading estimation using a smartphone placed in the user’s pant pocket. We also utilize PCA due to its success in local walking direction extraction. Compared with the PCA-based approach, RMPCA achieves more accurate estimation of the horizontal plane, since we obtain the vertical acceleration component in the projected reference coordinate system. Furthermore, we choose a specific reference coordinate system so that the local walking direction can be easily transformed into the global one without requiring noisy compass readings. Experimental results show that the proposed RMPCA approach outperforms the previous PCA and uDirect approaches in terms of accuracy and feasibility.

## 3. Overview

**Figure 1:**
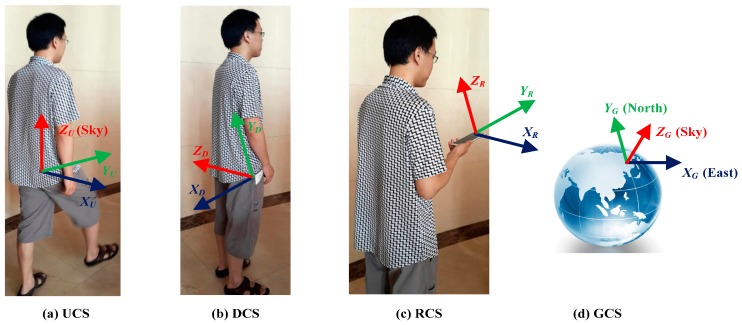
Illustration of the defined coordinate system: (**a**) UCS (**b**) DCS (**c**) RCS (**d**) GCS.

The user heading estimation is defined as the process of seeking the relative orientation of a user’s coordinate system (UCS) with respect to the global coordinate system (GCS). Let GCS be an Earth coordinate system defined by axes $X_{G}$, $Y_{G}$, and $Z_{G}$, which point east, north and the opposite direction of the gravity vector, respectively. As shown in Figure 1a, The UCS is defined by axes $X_{U}$, $Y_{U}$, and $Z_{U}$, with $Y_{U}$ being tangential to the trajectory, $Z_{U}$ coinciding with $Z_{G}$, $X_{U}$ being the right side of user body and given by the cross product of $Y_{U}$ and $Z_{U}$. In order to define the user’s orientation in GCS, we exploit the acceleration pattern generated during pedestrian walking. The inertial signals are measured on a smartphone with a three-axis accelerometer and a three-axis gyroscope built in, thus referring the third coordinate system called device coordinate system (DCS). DCS is defined by axes $X_{D}$, $Y_{D}$, and $Z_{D}$, where $X_{D}$ and $Y_{D}$ axes are parallel to phone screen and point rightward and forward, respectively, and $Z_{D}$ is given by the cross product of $X_{D}$ and $Y_{D}$. The GCS and DCS are depicted in Figure 1d and Figure 1b, respectively. The final coordinate system is the reference coordinate system (RCS), as shown in Figure 1c. RCS is the DCS at a specific moment when the user holds the smartphone in hand and initially starts the PDR application.

The proposed RMPCA mainly includes three steps. Firstly, acceleration signals measured at the DCS are projected into the RCS, by computing the related rotation matrix. Secondly, PCA over the horizontal plane of projected acceleration signals at RCS is applied for local walking direction extraction. Finally, the user’s local walking direction is transformed into a global one by a calibration process. There are three assumptions used in RMPCA. First, the smartphone is initially held and gazed at by the user for a few seconds, while the smartphone’s forward axis $Y_{D}$ is aligned with the user’s walking direction axis $Y_{U}$. This duration is always available, since the user needs to gaze at and manipulate the smartphone when he starts the application and sets related parameters. Second, the user’s initial orientation is assumed to be known as a *priori*, as in many other works [25,26]*.* This assumption is always reasonable, since the user’s continuous trajectories and related orientations can be initially obtained by several external positioning systems, such as Global Position System (GPS) tracking [25] when the user enters a building, WiFi localization [27] or landmarks [28]. Third, the user walks forward and relatively straight during a short period most of the time. Practically, since the user sways sideways while taking each step during straight walking, the walking direction is determined within a stride (*i.e.*, two adjacent steps). If the third assumption is invalid due to a user turn, we combine a turn detection algorithm to improve the heading estimation performance.

## 4. Methodology

### 4.1. Projection of Acceleration Signals into the RCS by Rotation Matrix

To extract the local walking direction, all the acceleration signals within a stride should be firstly projected into the horizontal plane. The PCA-based approach achieves this by deploying a recognized gravity vector. However, the DCS may vary a lot, even within one step, due to the leg rotational movements, thus rendering the recognized gravity vector at a certain DCS unusable directly. To adapt the changing DCS, we project all the acceleration signals into a RCS, and then apply the gravity vector at RCS to obtain the horizontal plane. To achieve this projection, we exploit the rotation matrix between DCS and RCS, since all inertial signals are measured at varying DCS. 

We develop an EKF-based attitude estimation model to compute the related rotation matrix. Instead of deploying Euler angles, we apply a rotation quaternion to describe the smartphone orientation, since it can avoid the singularity problems [29]. Firstly, we construct the relationship between a rotation quaternion and the smartphone orientation. The projection of acceleration signals at RCS into DCS can be represented as follows:

(1) $$a^{DCS}\left(t\right)={\left(C_{RCS}^{DCS}\left(q\left(t\right)\right)\right)}^{T}a^{RCS}\left(t\right)$$

where $C_{RCS}^{DCS}\left(q\left(t\right)\right)$ is the rotation matrix of DCS with respect to RCS at time t, $a^{RCS}\left(t\right)$ and $a^{DCS}\left(t\right)$ are the 3×1 acceleration vectors at time t relative to RCS and DCS. For the sake of simplicity, we will omit the argument t. The rotation matrix can be described by a quaternion:

(2) $$C_{RCS}^{DCS}\left(q\right)\text{=}\left[\begin{array}{ccc} q_{0}^{2}+q_{1}^{2}-q_{2}^{2}-q_{3}^{2} & 2\left(q_{1}q_{2}-q_{0}q_{3}\right) & 2\left(q_{1}q_{3}+q_{0}q_{2}\right)\\ 2\left(q_{1}q_{2}+q_{0}q_{3}\right) & q_{0}^{2}-q_{1}^{2}+q_{2}^{2}-q_{3}^{2} & 2\left(q_{2}q_{3}-q_{0}q_{1}\right)\\ 2\left(q_{1}q_{3}-q_{0}q_{2}\right) & 2\left(q_{0}q_{1}+q_{2}q_{3}\right) & q_{0}^{2}-q_{1}^{2}-q_{2}^{2}+q_{3}^{2} \end{array}\right]$$

where $q\text{= }{\left[\begin{array}{cc} q_{0} & \begin{array}{ccc} q_{1} & q_{2} & q_{3} \end{array} \end{array}\right]}^{T}$ is the normalized quaternion, $q_{0}$ is the scalar part of the quaternion and $\left[q_{1},q_{2},q_{3}\right]$ is the vector part. 

Secondly, according to the rigid body kinematic equations [29], the discrete-time model of rotation quaternions can be given as:

(3) $$q_{k+1}\text{ = exp}\left(0.5\times \Omega \left(w_{k}T_{\text{s}}\right)\right)q_{k}=\left(I\text{cos}\left(0.5\times \Delta \theta_{k}\right)+\Omega \left(w_{k}T_{\text{s}}\right)\text{sin}\left(0.5\times \Delta \theta_{k}\right)/\Delta \theta_{k}\right)q_{k}$$

where $T_{s}$ is the system interval, $q_{k}$ and $q_{k+1}$ are the quaternions at time instants $kT_{s}$ and $\left(k+1\right)T_{s}$ respectively, $w_{k}={\left[\begin{array}{ccc} w_{k}^{x} & w_{k}^{y} & w_{k}^{z} \end{array}\right]}^{T}$ is the angular velocity vector at time instants $kT_{s}$ relative to DCS, I is an 3×3 identity matrix, $\Delta \theta_{k}=T_{s}\sqrt{{\left(w_{k}^{x}\right)}^{2}+{\left(w_{k}^{y}\right)}^{2}+{\left(w_{k}^{z}\right)}^{2}}$, and $\Omega \left(w_{k}T_{s}\right)$ is given by:

(4) $$\Omega \left(w_{k}T_{s}\right)=T_{s}\left[\begin{array}{cccc} 0 & -w_{k}^{x} & -w_{k}^{y} & -w_{k}^{z}\\ w_{k}^{x} & 0 & w_{k}^{z} & -w_{k}^{y}\\ w_{k}^{y} & -w_{k}^{z} & 0 & w_{k}^{x}\\ w_{k}^{z} & w_{k}^{y} & -w_{k}^{x} & 0 \end{array}\right]$$

The quaternion $q_{k+1}$ is determined when the initial condition is set as $q_{0}={\left[\begin{array}{cccc} 1 & 0 & 0 & 0 \end{array}\right]}^{T}$. 

Finally, the EKF is applied to fuse the gyroscope data with the accelerometer data for device attitude estimation. We deploy the smartphone rotation quaternion as a state vector. The state transition vector equation can be given by:

(5) $$q_{k+1}\text{ = }F_{k}q_{k}+w_{k}^{q}$$

where the state transition matrix $F_{k}=\text{exp}\left(\Omega \left(w_{k}T_{\text{s}}\right)\right)$, and:

(6) $$w_{k}^{q}=\Xi_{k}w_{k}^{gyro}=-\frac{T_{s}}{2}\left[\begin{array}{c} {}[e_{k}\times ]+q_{0}^{k}I\\ -e_{k}^{T} \end{array}\right]w_{k}^{gyro}$$

$q_{k}\text{= }{\left[\begin{array}{cc} q_{0}^{k} & \begin{array}{ccc} q_{1}^{k} & q_{2}^{k} & q_{3}^{k} \end{array} \end{array}\right]}^{T}$ is the rotation quaternion at time instants $kT_{s}$, $q_{0}^{k}$ is the scalar part and $e_{k}\text{= }{\left[\begin{array}{ccc} q_{1}^{k} & q_{2}^{k} & q_{3}^{k} \end{array}\right]}^{T}$ is the vector part, $w_{k}^{gyro}$is the white Gaussian measurement noise vector for gyroscope at time instants $kT_{s}$, and $\left[e_{k}\times \right]$ is a standard vector cross-product operator. Equations (5) and (6) are derived from Equation (3), and can be considered as a first order approximation of the “noisy” transition matrix [30]. The approximation always performs well, since the gyroscope measurement noise vector $w_{k}^{gyro}$ is small enough in the application area. Consequently, the process noise covariance matrix $Q_{k}$ is given by:

(7) $$Q_{k}=w_{k}^{q}{\left(w_{k}^{q}\right)}^{T}=\Xi_{k}Q_{k}^{gyro}\Xi_{k}^{T}$$

where Qkgyro=σgyro2I is the covariance matrix for gyroscope measurement noise vector $w_{k}^{gyro}$.

The measurement model is constructed based on the acceleration signals observed when the human body is almost not affected by any acceleration:

(8) $$a_{k+1}\text{ = }{\left(C_{RCS}^{DCS}\left(q_{k+1}\right)\right)}^{T}g^{RCS}+v_{k+1}^{a}$$

where $a_{k+1}$ is the recognized gravity vector at DCS, $g^{RCS}$ is the gravity vector at RCS, and $v_{k+1}^{a}$ is the related white Gaussian measurement noise. Let $R_{k+1}$ be the covariance matrix of the measurement noise. To filter out the disturbance of significant human motions, we construct adaptive measurement noise covariance matrix $R_{k+1}={}^{R}\sigma_{a}^{2}I$:

(9) $${}^{R}{\text{σ}}_{a}^{2}=\{\begin{array}{cc} {\text{σ}}_{a}^{2}, & {\left\|a_{k+1}-g\right\|}_{2}<{\text{ε}}_{a}\\ \infty , & \text{otherwise} \end{array}$$

Thus, the measurement model can be approximated as a linearized formula:

(10) $$a_{k+1}\;=\;H_{k+1}q_{k+1}-H_{k+1}q_{k+1}^{-}+{\left(C_{RCS}^{DCS}\left(q_{k+1}^{-}\right)\right)}^{T}g+v_{k+1}^{a}$$

where $H_{k+1}={\partial a_{k+1}/\partial q_{k+1}|}_{q_{k+1}=q_{k+1}^{-},v_{k+1}^{a}=0}$ is the related Jacobian matrix, $q_{k+1}^{-}=F_{k}{\hat{q}}_{k}$ is the best state estimation of $q_{k+1}$ available, namely the *a priori* state estimate, ${\hat{q}}_{k}$ is the quaternion estimation result of EKF at time instants $kT_{s}$. 

Based on the state model in Equation (5) and the measurement model in Equation (10), with the process noise covariance matrix $Q_{k}$ and measurement noise covariance matrix $R_{k+1}$, the EKF for estimating the state vector $q_{k+1}$ may be established. Detailed procedures for executing the EKF may be found in [31]. Therefore, after estimating the state vector $q_{k+1}$, the projection of acceleration signals at DCS into RCS may be derived from Equation (1):

(11) $$a^{RCS}\left(t\right)=C_{RCS}^{DCS}\left(q\left(t\right)\right)a^{DCS}\left(t\right)$$

where q(t) is the related smartphone rotation quaternion at time t, $a^{RCS}\left(t\right)$ is the obtained projected acceleration signals at RCS. 

### 4.2. PCA for Local Walking Direction Extraction

**Figure 2:**
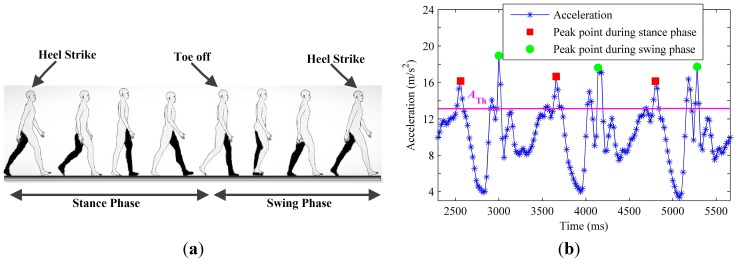
Acceleration patterns of pedestrian walking with the smartphone put in the pocket: (**a**) Walking cycle includes two phases: stance phase and swing phase; (**b**) The peak detection algorithm detects one stride with two footsteps (peak points).

PCA for walking direction extraction is based on the observation that the most variations in the horizontal plane will be parallel to the walking direction [20]. As shown in Figure 2a, the walking cycle of humans includes two main phases, the stance phase and the swing phase. The stance phase is usually defined as a period of the walking cycle, started with a heel strike moment and ended with a toe off moment [22]. Each phase corresponds to a footstep. As shown in Figure 2b, we empirically find that the norm of the acceleration signal is a robust feature for footstep detection. We deploy a peak detection algorithm [32,33] with two thresholds, which are used to eliminate false peaks caused by device shakes with too short time intervals or too small magnitude:

(12) $$\{\begin{array}{l} \Delta T\geq T_{\text{Th}}\\ \left|AccNorm-g\right|\geq A_{\text{Th}} \end{array}$$

where ΔT is the time interval between every two adjacent peaks, AccNorm is the norm of the three dimensional acceleration signal, g is the local gravity, $T_{\text{Th}}$ and $A_{\text{Th}}$ are the time threshold and the magnitude threshold for filtering false peaks, respectively. Therefore, each recognized peak point can stand a footstep. Moreover, as seen in Figure 2b, the peak point during the swing phase has a larger magnitude than that during the stance phase. This can be used to distinguish between the two phases within a stride. The peak detection algorithm may be applied successfully under most smartphone placements [33], such as in a hand or a pocket, though the two thresholds should be adjusted according to the different magnitude of acceleration signals. 

After recognizing a stride with the related acceleration signals, we obtain the horizontal plane at RCS by computing the related vertical component. We apply a sliding window over all three dimensions of the acceleration signals at the initial time when the smartphone is held initially by the user. If the variances of all dimensions are close to 0 and the total magnitude approaches 9.81 $\text{m}/{\text{s}}^{2}$, the signal is very likely to be dominated by the vertical component, and thus being recognized as the local gravity vector. Denote the gravity vector at RCS as the 3×1 vector $g^{RCS}$, the acceleration signals can be projected into the horizontal plane as given:

(13) $$a_{\text{hor}}^{RCS}\text{ = }a^{RCS}-{\left(g^{RCS}\right)}^{T}a^{RCS}g^{RCS}/{\left\|g^{RCS}\right\|}^{2}$$

where $a^{RCS}$ and $a_{\text{hor}}^{RCS}$ are the acceleration at RCS and the related horizontal component, respectively. 

**Figure 3:**
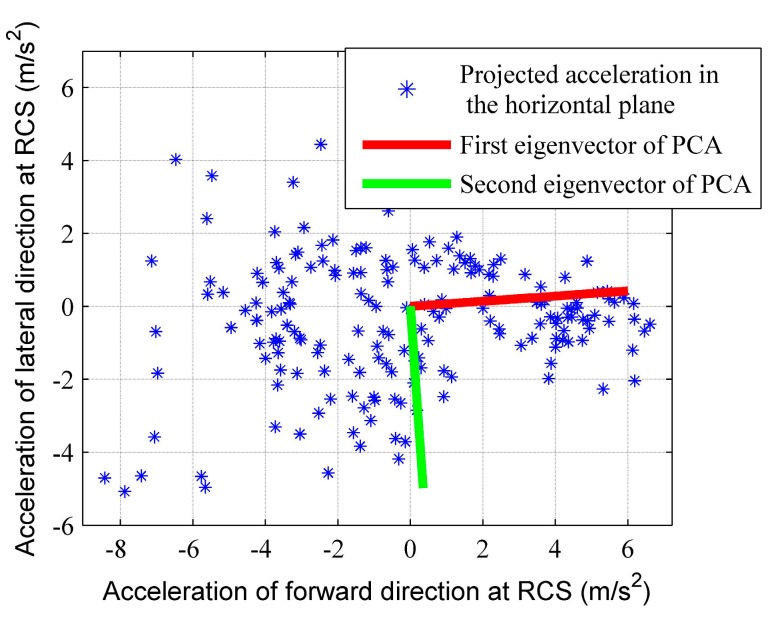
Illustration of PCA for eigenvector extraction over the horizontal plane of acceleration signals to infer the local walking direction at RCS.

**Figure 4:**
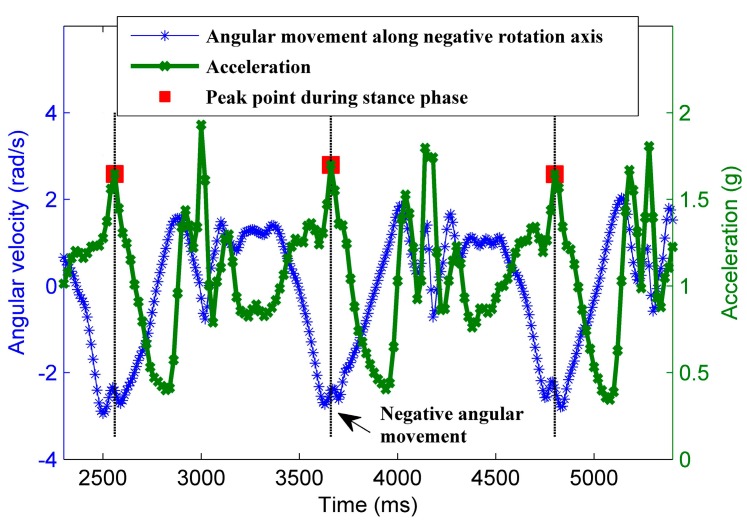
The rotation value along the negative (positive) direction of the rotation axis $X_{U}$ should be negative (positive) at the peak point during stance phase. This can be used to align the positive direction of the rotation axis $X_{U}$ to the right side of user body.

After obtaining the horizontal plane at RCS, PCA extracts the local walking direction by computing the related first eigenvector, as shown in Figure 3. However, PCA for walking direction extraction suffers from the 180° ambiguity problem. The obtained first eigenvector cannot distinguish between the forward and backward walking directions. To address this problem, we exploit the observation that, around the peak point during the stance phase, the thigh keeps swinging forward distinctly with the rotating smartphone. Thus, at that moment, there should be a positive (negative) rotation along positive (negative) direction of the rotation axis $X_{U}$. Figure 4 shows the angular velocity along the negative direction of the rotation axis, whose angular movement is negative at the peak point during the stance phase. After a rough estimation of the parallel direction of rotation axis $X_{U}$ as referred in [21], we can align the positive direction of rotation axis $X_{U}$ to the right side of the body by requiring the angular movement to be positive. Then, an approximation of the forward direction can be obtained by a rotation of 90° in the horizontal plane, and ultimately is used to eliminate the 180° ambiguity problem. 

### 4.3. Calibration Process for Determining Global Walking Direction

The local walking direction vector at RCS can be firstly projected into the UCS. Let $C_{UCS}^{RCS}$ be the rotation matrix of RCS with respect to UCS, $D^{RCS}$ be the local walking direction vector obtained at RCS. The walking direction vector at UCS can be given by:

(14) $$D^{UCS}=C_{UCS}^{RCS}D^{RCS}$$

where $D^{UCS}\text{=}{\left[\begin{array}{ccc} D_{x}^{UCS} & D_{y}^{UCS} & D_{z}^{UCS} \end{array}\right]}^{T}$ is the walking direction vector at UCS. Assume that RCS is subjected to three subsequent rotations, *i.e.*, a rotation by a pitch angle $\theta_{x}$ around $X_{U}$, a rotation by a roll angle $\theta_{y}$ around $Y_{U}$, and, finally, a rotation by a yaw angle $\theta_{z}$ around $Z_{U}$. The total rotation matrix can be obtained from three elementary rotation matrices:

(15) $$C_{UCS}^{RCS}=C_{z}\left(\theta_{z}\right)C_{y}\left(\theta_{y}\right)C_{x}\left(\theta_{x}\right)$$

(16) $$C_{x}\left(\theta_{x}\right)=\left[\begin{array}{ccc} 1 & 0 & 0\\ 0 & \text{cos}\theta_{x} & -\text{sin}\theta_{x}\\ 0 & \text{sin}\theta_{x} & \text{cos}\theta_{x} \end{array}\right]$$

(17) $$C_{y}\left(\theta_{y}\right)=\left[\begin{array}{ccc} \text{cos}\theta_{y} & 0 & \text{sin}\theta_{y}\\ 0 & 1 & 0\\ -\text{sin}\theta_{y} & 0 & \text{cos}\theta_{y} \end{array}\right]$$

(18) $$C_{z}\left(\theta_{z}\right)=\left[\begin{array}{ccc} \text{cos}\theta_{z} & -\text{sin}\theta_{z} & 0\\ \text{sin}\theta_{z} & \text{cos}\theta_{z} & 0\\ 0 & 0 & 1 \end{array}\right]$$

According to the first assumption in Section 3, the yaw angle $\theta_{z}$ at RCS is zero, since the smartphone’s forward axis $Y_{D}$ is aligned with the user’s walking direction axis $Y_{U}$. The unknown pitch angle $\theta_{x}$ and roll angle $\theta_{y}$ can be obtained by exploiting the local gravity vector as follows:

(19) $$g^{UCS}=C_{UCS}^{RCS}g^{RCS}$$

where $g^{UCS}={\left[\begin{array}{ccc} 0 & 0 & 9.81 \end{array}\right]}^{T}$ is the local gravity vector at UCS and $g^{RCS}$ is the measured gravity vector at RCS. After knowing the three angles, *i.e.*, the pitch angle $\theta_{x}$, the roll angle $\theta_{y}$, and the yaw angle $\theta_{z}$, the rotation matrix $C_{UCS}^{RCS}$ can be computed by Equation (15). 

According to the second assumption, we assume that the initial yaw angle of the user orientation at GCS is $\psi_{0}$. Finally, the global walking direction, i.e. the yaw angle ψ at GCS, can be given as:

(20) $$\psi =\text{arctan}\left(\frac{D_{y}^{UCS}}{D_{x}^{UCS}}\right)+\psi_{0}-\frac{\pi }{2}$$

### 4.4. Turn Detection Algorithm for Improving RMPCA

**Figure 5:**
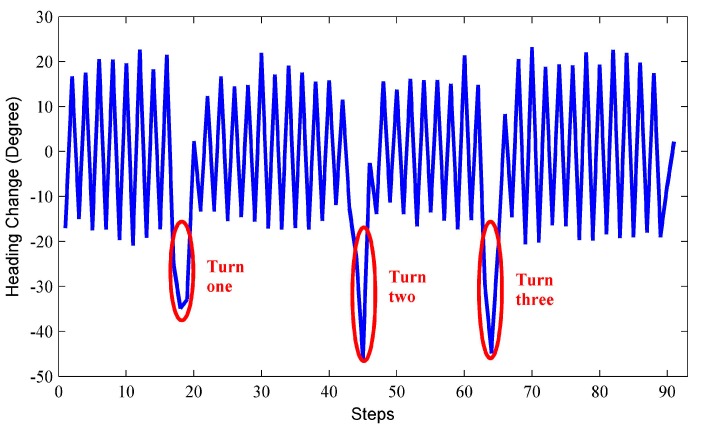
Identification of turns.

The basic idea of turn detection is to exploit the heading change pattern during pedestrian walking. The user heading changes alternative between positive and negative with the similar amplitude when pedestrian walks straight and sways sideways, as shown in Figure 5. If the heading change pattern is corrupted to some degree, a user turn is detected. We deploy the gyroscope outputs in horizontal plane to compute the heading change for each step. Derived from Equations (11) and (14), the angular velocity at UCS can be given by related rotation matrix:

(21) $$w^{UCS}\left(t\right)=C_{UCS}^{RCS}C_{RCS}^{DCS}\left(q\left(t\right)\right)w^{DCS}\left(t\right)$$

where q(t) is the related rotation quaternion at time t, $w^{UCS}\left(t\right)$ and $w^{DCS}\left(t\right)$ are the representations of angular velocity signal at UCS and DCS at time t respectively, $C_{UCS}^{RCS}$ is the rotation matrix of RCS with respect to UCS, and $C_{RCS}^{DCS}\left(q\left(t\right)\right)$ is the rotation matrix of DCS with respect to RCS at time t. Therefore, the heading change for each step can be given by:

(22) $$\delta \theta_{i}=\sum_{k=1}^{N_{i}}w_{k,i}^{UCS}\left(Z_{U}\right)T_{gyro}$$

where $\delta \theta_{i}$ is the heading change for step i, $w_{k,i}^{UCS}\left(Z_{U}\right)$ is the angular velocity component rotating around $Z_{U}$ of the k−th angular velocity sample for step i, $N_{i}$ is the total number of samples within the step i, and $T_{gyro}$ is the related sampling interval of gyroscope outputs.

After computing the heading change for each step, if we find a positive (negative) heading change follows a positive (negative) one, and the absolute heading change of the two steps exceeds a given threshold $\delta \theta_{\text{th}1}$, the heading change pattern during straight walking is considered to be corrupted. As a result, a user turn is reported. Therefore, we estimate the global walking direction by adding the current step heading change on the heading of previous step. Inversely, if a positive (negative) heading change follows a negative (positive) one, and the absolute heading change is smaller than a given threshold $\delta \theta_{\text{th2}}$, we recognize that the user turn terminates and deploy RMPCA again.

## 5. Evaluation

### 5.1. Experimental Setup

**Figure 6:**
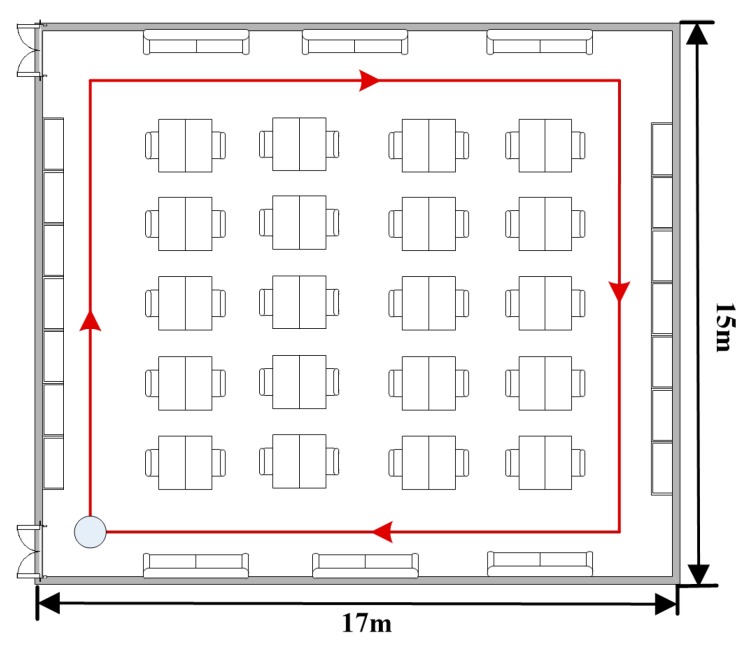
Indoor test environment.

We have tested our heading estimation approach in an office building, as shown in Figure 6. Six subjects with different physical characteristics were asked to walk along the path denoted by a red solid line in Figure 6. The size of the walking path is 14.4 m × 12 m with a total length of 52.8 m, which requires an average of 88 steps (44 strides) to complete. Each subject firstly held the phone in hand and stands for few seconds to start the application. Then, the subject put the phone into the pocket and walked along the path. The subjects were free to change the device orientation arbitrarily when he put the phone into the pocket. Each subject repeated the above procedure at least 10 times. Thus, 5280 samples could be collected for the evaluation of the proposed approach. To label the ground truth, the subjects were asked to walk along the densely placed tags on the ground carefully. We also used a camera to record the entire walking procedure. Before carrying out experiments, we did necessary calibrations to make the gyroscope outputs more precise and robust. As in many other literatures [25,26] do, the user’s initial state, including location and orientation, is assumed to be known as a *priori*.

The experiments were performed indoors, where severe magnetic perturbations existed and were difficult to calibrate. Thus, the compared approaches PCA and uDirect adopted outdoors could not deploy a compass to transform the local walking direction into the global one. For convenience of comparisons, the local walking directions obtained by the PCA and uDirect approaches were also transformed into the global one by our calibration process. Therefore, in our experiments, the accuracy performance differences of the compared approaches were mainly caused by their local walking direction extraction schemes.

### 5.2. Heading Estimation Performance Analysis

**Figure 7:**
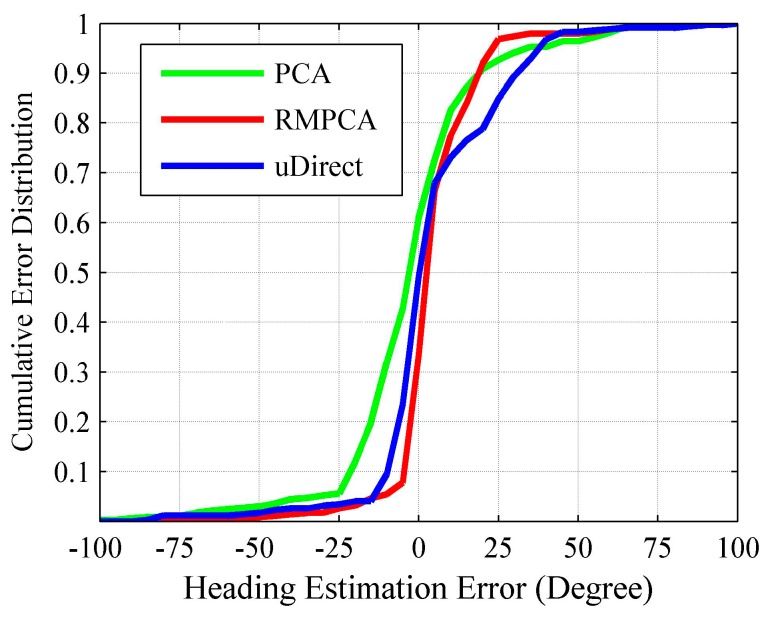
Heading estimation error distribution.

**Figure 8:**
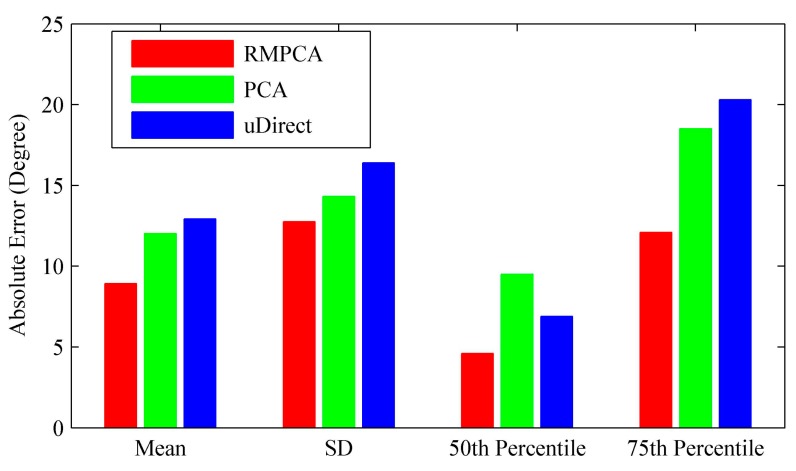
Performance comparisons of the absolute heading estimation error: mean, standard deviation (SD), 50th percentile and 75th percentile.

**Figure 9:**
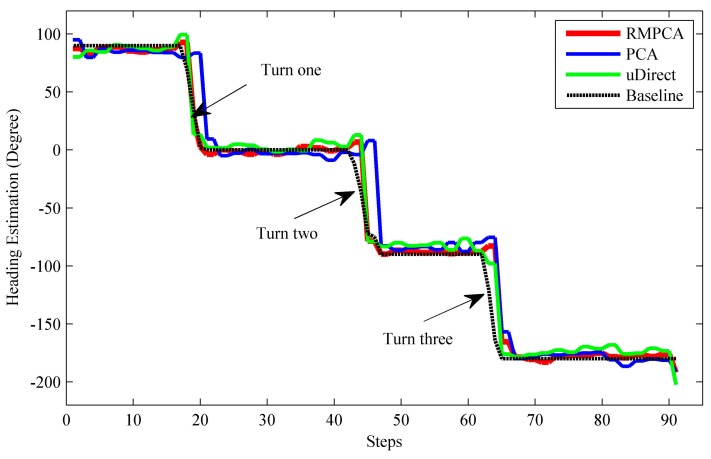
Heading estimations of different approaches vs. baseline.

Figure 7 compares the heading estimation error distributions of different approaches, respectively. Clearly, RMPCA performs best, since the estimation errors distributed in the large magnitude field are much less than those of the other two approaches. Similar results can be seen in Figure 8. The 50th percentile absolute heading estimation errors of RMPCA, PCA and uDirect are 4.6°, 9.5°, and 6.9°, respectively, while the 75th percentile absolute errors are 12.1°, 18.5° and 20.3°. RMPCA reduces the mean absolute estimation error by 25.8% (3.1°), and 31.0% (4.0°) than PCA and uDirect, respectively. Figure 9 shows the heading estimation results of the compared approaches for one trace. One can clearly see that the heading estimation of our RMPCA is the closest to the baseline direction.

The main difference between PCA and our RMPCA is the estimation scheme of the vertical acceleration components, which is used to infer the horizontal plane of acceleration signals. PCA obtains the vertical components by finding acceleration signals whose magnitude approaches $\text{9.81 }\text{m}/{\text{s}}^{2}$ and the variance of three dimensions is close to 0. However, this scheme suffers from two drawbacks. First, the obtained vertical components are always suboptimal due to the noisy components introduced by walking locomotion. Second, the DCS of the obtained vertical component does not coincide with the DCS of the other acceleration signals within the same stride. Thus, directly deploying the obtained vertical component to compute the horizontal plane renders an inaccurate estimation. In contrast to the PCA approach, our RMPCA approach avoids the second drawback by projecting the acceleration signals into the same RCS. For the first drawback, the noisy components introduced by the body locomotion will be alleviated substantially by choosing the RCS when users stand and start the application for few seconds. Therefore, our RMPCA approach performs much better than the PCA approach. 

For the uDirect approach, the local walking direction is extracted at the moment when the side-to-side acceleration component is minimized during the walking cycle. Unfortunately, such a moment is susceptible to sideway acceleration components. Even when the side-to-side acceleration component is indeed minimized, the walking direction component may not dominate the horizontal plane of acceleration signals, according to our experimental study. Therefore, uDirect approach performs the worst and is more likely to generate heading estimation errors of large magnitude.

### 5.3. Turn detection for Heading Estimation Improvement

**Figure 10:**
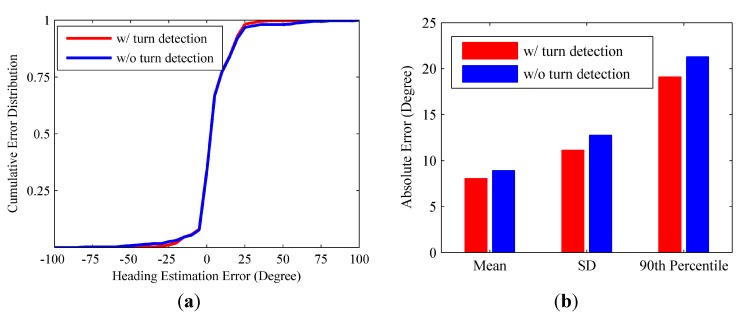
Heading estimation performance comparisons between RMPCA with and without turn detection. (**a**) Error distribution; (**b**) Statistical results of the absolute error.

If the user is making a turn, the proposed RMPCA and compared approaches all suffer from degraded performance. This may be contributed to the substantial sideway acceleration jitters during the user turn, which corrupt the acceleration pattern exploited by the heading estimation approaches. As seen in Figure 9, the estimated headings all deviate from the baseline headings significantly when the user makes three turns. Therefore, we deploy the turn detection algorithm proposed in Section 4.4 to improve the heading estimation. Once a turn is reported, we estimate the current heading for each step by adding the current heading change in Equation (21) to the heading of previous step. The heading change scheme for heading estimation performs relatively well during a short period, while estimation error may accumulate rapidly due to the jitters caused by body locomotion. Thus, if a turn termination is recognized, we deploy the proposed RMPCA for heading estimation again. Figure 10 shows the heading estimation performance improvement of RMPCA by combing the proposed turn detection algorithm. Since the estimation errors of large magnitude caused by the user turns are reduced, the tail of the error distribution is cut down. Particularly, the turn detection algorithm reduces the mean absolute error, standard deviation (SD), 90th percentile from 8.93°, 12.75°, and 21.3° to 8.05°, 11.12°, and 19.1°, respectively.

### 5.4. PDR Application

**Figure 11:**
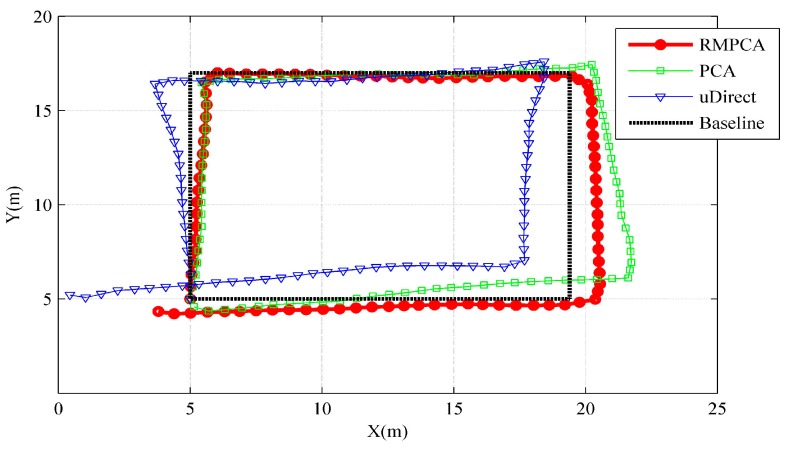
Tracking performance comparisons of one trace between different heading estimation approaches.

Although the heading estimation for PDR using a smartphone under a highly controlled situation, such as held in hand, has been widely used, it is still an unsolved problem for smartphones placed in the user’s pant pocket. The proposed RMPCA aims to solve this problem by three steps: project the acceleration signals into RCS by a related rotation matrix, then estimate the local walking direction at RCS by applying PCA, and finally obtain the global walking direction at GCS by a calibration process. In order to calculate the walking distance, a step length estimation model should also be developed. Though the step lengths are determined by various factors, including height, attitude, and walking frequency [16,33], for the same pedestrian, it mainly depends on the walking speed. For different pedestrians, we may develop a memorization function to store the personal parameters of step length estimation model. Considering the strong correlation of the walking speed with the amplitude of the measured acceleration, we deploy the empirical model [34] given as follows:

(23) $$StepLength=K\sqrt[{4}]{Acc_{\text{max}}\text{−}Acc_{\text{min}}}$$

where $Acc_{\max}$ and $Acc_{\min}$ represent the maximum and minimum amplitudes of vertical acceleration components during each stride, K is the personalized parameter that need to be calibrated for each pedestrian. Figure 11 shows the tracking trajectories comparisons of one trace between different heading estimation approaches. Clearly, the heading estimation error is the main localization error source for PDR. Thus, the proposed RMPCA with the smallest heading estimation error obtains the best tracking performance. As shown in Table 1, the proposed RMPCA are the only approach whose mean error and 50th percentile error are within 1.5 m. RMPCA reduces the mean localization error by 32.2% (0.66 m), and 37.7% (0.84 m) than that of PCA and uDirect, respectively.

**Table 1:** Comparisons of real-time localization errors (m).

Heading Estimation Approaches	Real-Time Localization Errors			
	Mean	Standard Deviation	50th Percentile	95^th^ Percentile
**RMPCA**	1.39	1.16	1.43	3.58
**PCA**	2.05	1.62	2.14	5.04
**uDirect**	2.23	1.84	2.30	5.67

## 6. Conclusions and Future Work

This paper presents RMPCA, an approach to determine pedestrian walking direction indoors using a smartphone placed in the user’s pant pocket, independent of the smartphone’s orientation. First, we develop an EKF-based attitude estimation model to compute the rotation matrix between DCS and RCS, which is used to adapt the changing DCS and obtain the horizontal plane acceleration signals at RCS. Then, PCA is applied over the horizontal plane for local walking direction extraction. Finally, we develop a calibration process for global walking direction translation without requiring noisy compass readings, which are almost unavailable in most indoor environments. Besides, a user turn detection algorithm exploiting heading change pattern is used to improve the heading estimation. Experimental results show that our approach reduces the mean absolute heading estimation error by 25.8% (3.1°), and 31.0% (4.0°) compared that of PCA and uDirect, respectively. 

In future works, to verify the effectiveness of the proposed RMPCA, we will perform the experiments at more complicated conditions, such as on various curved walking paths and with random walking velocities. The effect of other smartphone mounting positions, including in a bag, against the ear during phone calling and in a swinging hand, will also be studied for the proposed RMPCA. More importantly, we will further expand the proposed heading estimation approach to the long term indoor navigation application, which has been paid few attentions. The main challenge is the accumulated error caused by gyroscope biases. We will introduce some landmarks or building map information for gyroscope bias calibrations and related orientation self-calibrations. Besides, some external localization techniques such as Wi-Fi fingerprinting and RFID may be combined to provide the initial user position and orientation estimations.
